# Bilateral Lower Extremity Chronic Exertional Compartment Syndrome With Recurrent Symptoms After Prior Fasciotomy: A Case Report

**DOI:** 10.7759/cureus.115995

**Published:** 2026-09-09

**Authors:** Thomas Shelton, Jake Ayisi, Mohamed S Ahmed, Taneja Rishabh

**Affiliations:** 1 Family Medicine, Champlain Valley Physicians Hospital, Plattsburgh, USA; 2 Physical Medicine and Rehabilitation, University of Vermont Larner College of Medicine, Burlington, USA

**Keywords:** chronic exertional compartment syndrome (cecs), exertional compartment syndrome, sport and exercise medicine, sports overuse injuries, student athletes

## Abstract

Chronic exertional compartment syndrome (CECS) is an exercise-related cause of lower extremity pain characterized by reproducible pain, tightness, cramping, and neurologic symptoms that improve with rest. Diagnosis may be delayed because chronic lower leg pain has a broad differential diagnosis, and currently used diagnostic strategies remain imperfect. We report the case of a young woman who began experiencing bilateral lower extremity pain during high school sports that progressed to pain with prolonged standing. Treadmill-provoked bilateral four-compartment pressure testing reproduced severe symptoms and demonstrated elevated pressures in multiple compartments after ten minutes of exertion, supporting the diagnosis of bilateral lower extremity CECS. She subsequently underwent bilateral four-compartment fasciotomy with initial symptom improvement. Several years later, she presented again with recurrent symptoms, new right ankle swelling, and neurologic findings that broadened the differential diagnosis beyond recurrent CECS. This case highlights the value of pressure testing in the initial diagnosis of CECS, the possibility of persistent or recurrent symptoms after fasciotomy, and the importance of reconsidering vascular and neurologic alternatives when patients return with symptoms after prior decompression.

## Introduction

Chronic exertional compartment syndrome (CECS) is an under-recognized but clinically important source of exercise-induced lower extremity pain [1-3]. During exercise, increased compartmental pressures and swelling reduce blood flow, leading to ischemia, discomfort, and neurological changes. Patients typically report predictable aching, tightness, cramping, weakness, or paresthesias with activity and improve following cessation of the provoking exercise [1,2]. The lower leg is the most commonly involved site. Symptoms often overlap with those of other causes of exertional leg pain, potentially delaying diagnosis [2,4].

The differential diagnosis for chronic lower leg pain includes medial tibial stress syndrome (diffuse periosteal pain along the tibial shaft with impact activity), stress fracture (focal bony tenderness with imaging correlation), nerve entrapment (sensory or motor deficits in a nerve territory), popliteal artery entrapment syndrome (exertional calf claudication caused by popliteal artery compression with vascular findings on provocative testing), lumbar radiculopathy (pain and neurologic symptoms following a nerve root distribution), and other vascular or myofascial conditions [4-6]. Intracompartmental pressure testing continues to be used in addition to the history and physical examination, although the optimal protocol and threshold for interpretation remain debated [3,7]. We present a case of bilateral lower extremity CECS supported by treadmill-provoked pressure testing in a young woman who later returned with recurrent symptoms after prior bilateral fasciotomy, emphasizing both the diagnostic pathway to CECS and the need to broaden the differential diagnosis when postoperative symptoms recur.

## Case presentation

A 25-year-old woman presented for reevaluation of bilateral lower leg pain after a prior diagnosis of CECS and a four-compartment bilateral fasciotomy. Detailed operative notes were unavailable, but medical records and the patient confirmed a four-compartment release. The onset of her original symptoms began during adolescence while playing high school sports, with bilateral leg pain that was worse on the right. By her twenties, symptoms were also provoked by prolonged standing at work. She described predictable anterior lower-leg pain after approximately two hours on her feet, occasional numbness involving all toes, discomfort when her legs were bumped, and episodes that carried into the following day. Prior physical therapy, shoe modifications, and orthotic use had not provided meaningful symptomatic relief.

Physical examination during the initial orthopedic workup demonstrated bilateral tibial tenderness, greater on the right, including discomfort with percussion and varus bending. Plain radiographs showed no evidence of stress injury. MRI of the right lower extremity showed no stress fracture, periostitis, or cortical thickening. The treating surgeon incidentally noted relatively decreased signal in the right anterior compartment compared with the left on anteroposterior images, but the clinical significance of this finding remained uncertain. Due to these symptoms being chronic, functionally limiting, and not explained by structural imaging, she was referred for compartment pressure testing.

She underwent bilateral lower leg compartment pressure testing approximately one year after symptom escalation. At that visit, she reported several years of cramping and tight pain previously labeled as shin splints, toe tingling that improved with rest, and symptoms that had occurred during high school sports and continued during prolonged standing at work. Pre-exercise pressures were measured in all four compartments of both lower legs. Needles were placed via anatomical landmarks. The device make and model were not reported. She then ran on a treadmill for 10 minutes until severe bilateral leg pain was reproduced at 8/10 intensity, after which immediate post-exercise pressures were obtained. Table 1 summarizes the pressure values. Because one-minute and five-minute post-exercise pressures were not reported, a diagnosis could not be strictly made via Pedowitz criteria. However, several pre-exercise pressure values were high, and immediate post-exercise pressures were elevated after reproduction of her typical symptoms. Based on the pressure profile and clinical symptom reproduction, the treating physician concluded that the findings were consistent with bilateral CECS and recommended consultation regarding bilateral four-compartment fasciotomy.

**Table 1:** Bilateral lower leg compartment pressure testing performed at the initial evaluation. The patient ran on a treadmill for 10 minutes until severe bilateral leg pain was reproduced at an intensity of 8/10, after which immediate post-exercise measurements were obtained.

Compartment	Left pre-exercise (mmHg)	Left immediate post-exercise (mmHg)	Right pre-exercise (mmHg)	Right immediate post-exercise (mmHg)
Anterior	15	70	28	33
Lateral	19	30	18	33
Superficial posterior	29	38	21	58
Deep posterior	14	62	16	26

She underwent a bilateral lower leg four-compartment fasciotomy at an outside hospital approximately three months after pressure testing. She reported meaningful initial symptom improvement, with resolution of exercise-provoked leg pain and a return to normal daily activity in the months following surgery. The complete outside operative report was not available, limiting detailed analysis of the exact surgical approach, intraoperative findings, and early postoperative rehabilitation.

Approximately six years after surgery, she returned, reporting a multi-month history of recurrent lower extremity discomfort. Her clinical picture was characterized by persistent tightness and frequent episodes of calf cramping, described as *charley horses*, alongside newly developed edema of the right ankle; notably, these findings were more severe on the right and worsened with activity. This was somewhat consistent with her prior presentation, raising concern for recurrence, particularly given her highly active job. Furthermore, she noted persistent postoperative sensory deficits localized to the medial right lower extremity, extending to the medial malleolus. Her occupational demands involved full-time employment requiring long periods of standing, and her current self-management strategies included thermotherapy and the as-needed use of acetaminophen and ibuprofen. At the time of re-presentation, she was not utilizing bracing and had not initiated a structured physical therapy regimen. 

Examination at the follow-up visit demonstrated well-healed bilateral fasciotomy scars and compartments that initially felt soft. Sensation in an L5 distribution was decreased on the right compared with the left. There was subtle right-sided weakness in an L4-L5 distribution, graded 4+/5, while the left side remained intact. Calf discomfort with passive dorsiflexion was more pronounced on the right. The remainder of the neurovascular examination was intact. No new imaging was available at that visit. After discussion of recurrent exertional compartment syndrome symptoms and the possibility of alternative causes, the treating surgeon referred her to physical therapy and planned follow-up in two months. If symptoms persisted, the plan was to evaluate for vascular causes, including popliteal artery entrapment syndrome, and to assess for radiculopathy with electromyography. Given the wide range of possible etiologies and the patient's nonspecific symptoms, ruling out alternative causes was prioritized before repeating compartment pressure measurement or considering further surgical intervention.

The overall clinical timeline is shown in Figure 1.

**Figure 1:**
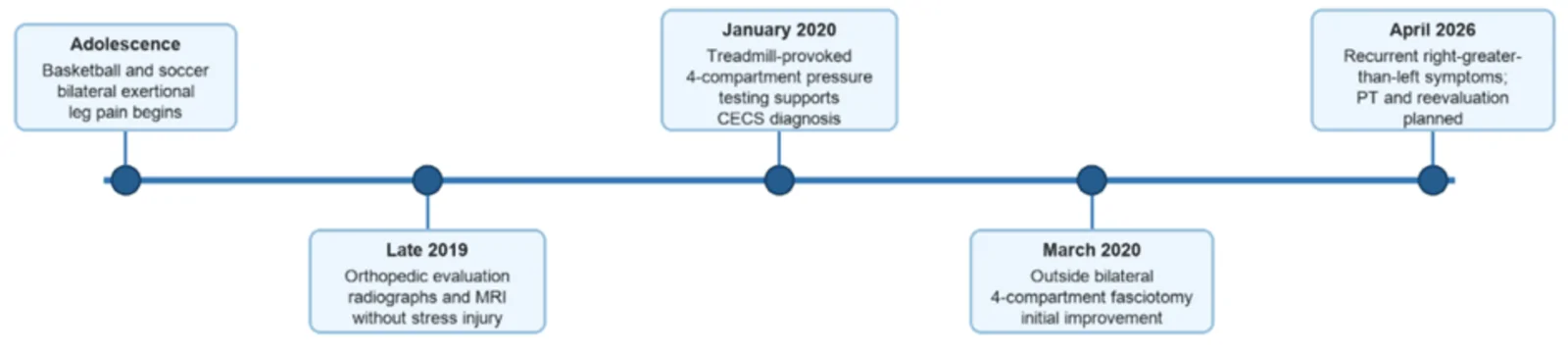
Clinical timeline of bilateral lower extremity CECS and post-fasciotomy recurrent symptoms. Author-created clinical timeline summarizing the course of bilateral lower-extremity chronic exertional compartment syndrome (CECS), outside fasciotomy, and later recurrent symptoms requiring reassessment. Image created by the authors using Google Docs software (Alphabet Inc., Mountain View, CA).

## Discussion

This case illustrates the diagnostic complexity of CECS and the importance of maintaining a broad differential diagnosis in patients with chronic lower leg pain. Before compartment testing, this patient underwent evaluation for stress injury with radiographs and an MRI that did not identify a structural bony explanation. That sequence is consistent with the broader CECS literature, in which patients often undergo prolonged workup for more common causes of exertional leg pain before pressure testing is pursued [2,4].

Her initial presentation was clinically compatible with CECS: symptoms were chronic, bilateral, reproducible, and improved with rest. Pressure testing reproduced severe symptoms and showed elevated immediate post-exercise values in multiple compartments, with several pre-exercise values also meeting the Pedowitz threshold. The Pedowitz criteria define a positive study as a resting pressure of 15 mmHg or higher, a pressure of 30 mmHg or higher at one minute after exercise, or a pressure of 20 mmHg or higher at five minutes after exercise in any compartment. These thresholds remain widely cited, but more recent reviews have emphasized ongoing uncertainty surrounding optimal testing protocols and the limitations of currently available diagnostic strategies [3,7]. In the present case, the pressure study was clinically useful because it was performed in the setting of a strongly suggestive history and otherwise unrevealing imaging.

The most educational feature of this case is the diagnostic uncertainty posed by recurrent symptoms following a chart-documented bilateral fasciotomy. Fasciotomy remains the standard operative treatment for appropriately selected patients with CECS, but published outcomes are variable, and persistent or recurrent symptoms are well described [8-10]. Reported recurrence rates after fasciotomy vary considerably across studies, ranging from as low as 0.65% in some systematic reviews to as high as 36% to 45% in select cohorts. The large range appears to be primarily driven by studies of military populations, who generally report higher recurrence rates than civilian athletes [8,9]. Systematic reviews have demonstrated heterogeneity in operative techniques, the number of compartments released, and outcome reporting, making recurrence rates difficult to compare directly across studies [8,9]. Outcome studies nevertheless suggest that although many patients improve after surgery, some patients experience incomplete recovery, ongoing symptoms, or the need for revision evaluation [9,10].

Importantly, recurrence should not automatically be assumed to represent reconfirmed recurrent CECS. Recurrent exertional pain after fasciotomy may reflect recurrent CECS, incomplete release, postoperative scarring, altered mechanics, or another pain generator entirely. In this patient, new right ankle swelling and right-sided sensory and motor findings in an L4-L5/L5 distribution broadened the differential diagnosis beyond compartment syndrome alone. Vascular causes, especially functional popliteal artery entrapment syndrome, may mimic CECS and have been specifically identified in the literature as a source of diagnostic confusion and potentially unnecessary surgery [5,6]. PAES occurs in approximately 0.17% to 3.5% of the general population and primarily affects young athletes; it presents with exertional calf claudication that resolves with rest and can be distinguished from CECS by diminished or absent distal pulses on provocative plantar- or dorsiflexion testing and vascular imaging demonstrating popliteal artery compression [5,6]. Likewise, lumbar radiculopathy or peripheral nerve pathology remains important when neurologic findings do not localize cleanly to a compartment syndrome alone [1,2]. Nonsurgical management options for CECS recurrence include activity modification, gait and foot-strike retraining, and botulinum toxin injection, although the evidence base for these approaches remains limited primarily to case series, and they are less consistently effective than primary fasciotomy [2,3].

This report has limitations. The outside operative note from 2020 was not available for direct review, so the exact decompression technique, number and extent of released compartments, intraoperative findings, and early postoperative rehabilitation could not be confirmed. Even with these limitations, the case remains instructive because it demonstrates both a well-documented objective diagnosis of bilateral lower extremity CECS and the diagnostic caution required when symptoms recur after surgery.

## Conclusions

This case describes bilateral lower extremity CECS supported by treadmill-provoked four-compartment pressure testing in a young woman with years of activity-related and standing-related leg pain. After chart-documented initial improvement following an outside bilateral four-compartment fasciotomy, she later re-presented with recurrent symptoms and new neurologic and swelling features that broadened the differential diagnosis. This case underscores two practical lessons: pressure testing can be highly useful when the history strongly suggests CECS, and postoperative recurrence should prompt careful reassessment for vascular and neurologic alternatives before further invasive intervention is considered.
